# Ozone Tolerance Found in *Aegilops tauschii* and Primary Synthetic Hexaploid Wheat

**DOI:** 10.3390/plants8070195

**Published:** 2019-06-28

**Authors:** Clare Brewster, Felicity Hayes, Nathalie Fenner

**Affiliations:** 1 Centre for Ecology & Hydrology, Environment Centre Wales, Bangor LL57 2UW, UK; 2 School of Natural Sciences, Bangor University, Bangor LL57 2UW, UK

**Keywords:** air pollution, *Triticum aestivum* L., *Triticum urartu*, *Triticum dicoccoides*, wheat, wild relatives

## Abstract

Modern wheat cultivars are increasingly sensitive to ground level ozone, with 7–10% mean yield reductions in the northern hemisphere. In this study, three of the genome donors of bread wheat, *Triticum urartu* (AA), *T. dicoccoides* (AABB), and *Aegilops tauschii* (DD) along with a modern wheat cultivar (*T. aestivum* ‘Skyfall’), a 1970s cultivar (*T. aestivum* ‘Maris Dove’), and a line of primary Synthetic Hexaploid Wheat were grown in 6 L pots of sandy loam soil in solardomes (Bangor, North Wales) and exposed to low (30 ppb), medium (55 ppb), and high (110 ppb) levels of ozone over 3 months. Measurements were made at harvest of shoot biomass and grain yield. *Ae. tauschii* appeared ozone tolerant with no significant effects of ozone on shoot biomass, seed head biomass, or 1000 grain + husk weight even under high ozone levels. In comparison, *T. urartu* had a significant reduction in 1000 grain + husk weight, especially under high ozone (−26%). The older cultivar, ‘Maris Dove’, had a significant reduction in seed head biomass (−9%) and 1000 grain weight (−11%) but was less sensitive than the more recent cultivar ‘Skyfall’, which had a highly significant reduction in its seed head biomass (−21%) and 1000 grain weight (−27%) under high ozone. Notably, the line of primary Synthetic Hexaploid Wheat was ozone tolerant, with no effect on total seed head biomass (−1%) and only a 5% reduction in 1000 grain weight under high ozone levels. The potential use of synthetic wheat in breeding ozone tolerant wheat is discussed.

## 1. Introduction

Bread wheat, *Triticum aestivum* L., is a very adaptable crop plant, successfully growing in a wide variety of environments and climatic conditions across the globe. This adaptability is due in part to the complexity of its genome, derived from its progenitor diploid and tetraploid species [1,2] and containing extensive gene replication and diversity [3]. However, the stagnation in the growth of global wheat yields over the last twenty-five years has raised concern because of wheat’s central role in global food systems and growing pressures on food security [4]. Considerable efforts are being made to increase wheat yields but many abiotic stress factors also continue to suppress yields, including ground level ozone pollution [5,6,7,8].

Ozone is created by photochemical reactions between sunlight and precursor gases including nitrogen oxides and volatile organic compounds [9]. Background levels of 25–55 ppb often occur, especially in the northern hemisphere where the highest concentrations of anthropogenic emissions of these precursor gases are found [10]. Although peak ozone episodes above 100 ppb are now less frequent in Europe and North America, they are still a common occurrence in East Asia [11,12]. Wheat is an ozone sensitive species and current levels of ground level ozone suppress mean global wheat yields in the northern hemisphere by 7.1–9.9% per year [6,13].

Ozone enters plants through their stomata, and in common with other types of abiotic stress, leads to oxidative stress through the creation of excess reactive oxygen species that oxidize proteins, DNA, RNA and lipids and may also cause the degradation of cell membranes [14] (pp. 737–738), all of which lead to reduced photosynthesis, early senescence and, ultimately, reduced seed production [15].

### 1.1. Differences in Ozone Sensitivity and Tolerance

Spring and winter wheat types are equally affected [15], with the growth phases between anthesis and the end of grain-fill found to be the most sensitive [16,17]. Studies researching the effects of ozone on different wheat cultivars have found some exhibiting greater tolerance to ozone than others, both above- [18,19] and below-ground [20]. In both spring wheat [21,22] and winter wheat [23] the more recent the cultivar the more ozone sensitive it has been found to be, though this is not always the case [24]. What causes the different responses between tolerant and sensitive cultivars, and to what extent this is directly attributable to factors such as higher stomatal conductance increasing ozone flux into the leaf, antioxidant enzymes enabling detoxification, or plant-soil-microbial interactions which act to mitigate negative effects, is still under investigation [17,25,26].

### 1.2. Role of Wild Relatives and Synthetic Wheat in Wheat Breeding

**Figure 1:**
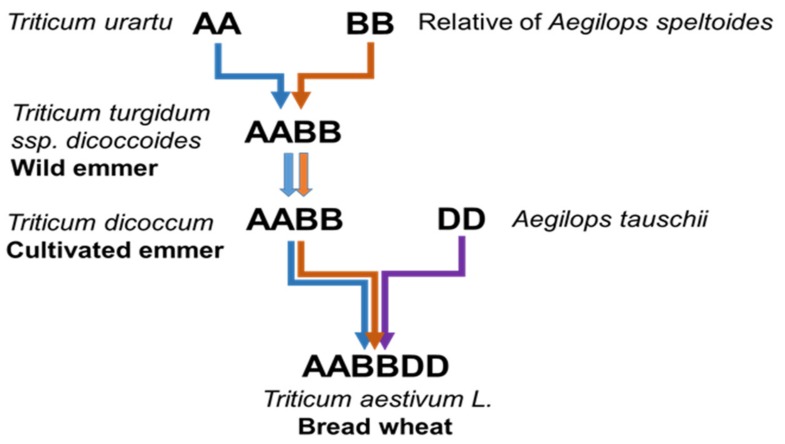
Phylogenetic history of *Triticum aestivum* L. showing the hybridisation events between *Aegilops* and *Triticum* species which led to the creation of bread wheat (after Marcussen et al., 2014).

A series of hybridisation events between *Triticum* and *Aegilops* species (Figure 1) led to the creation of hexaploid bread wheat c. 8000 years ago [2]. With the genomes of wheat and its closest wild relatives now sequenced, there is great potential for wheat improvement [1,27,28,29] as the diversity found within wheat’s wild relatives is recognised as a valuable source of biotic and abiotic tolerance [30,31].

Assessments of the ozone sensitivity of some of the close wild relatives of wheat have been made previously in short term experiments [32,33], with young plants (3–6 weeks old) receiving 3 weeks of ozone fumigation at 100 ppb concentrations. *Ae. tauschii* (diploid DD genome donor) appeared ozone sensitive, with reductions in shoot, root and total biomass and concomitant reductions in physiological performance. The diploid AA genome, *T. monococcum*, was also found to be sensitive [32], though less so than *Ae. tauschii,* and therefore *Ae. tauschii* was suggested as the likely genetic origin of wheat’s ozone sensitivity [32]. Assessments of tetraploid wheats, *Triticum turgidum* ssp. *durum* [32] and *T. dicoccoides* [33] suggested they had greater ozone tolerance.

Synthetic Hexaploid Wheat, or resynthesized wheat, is a modern artificial re-crossing of the tetraploid AABB and diploid DD species [34] with the majority of recent synthetic wheat lines being created from crosses between lines of *T. turgidum* ssp. *durum*, (AABB genome) and accessions of *Ae. tauschii* [35]. The resulting primary synthetic wheat lines are now used in wheat breeding because they contain a wealth of wild relative genetic diversity lost during wheat domestication, and are also readily crossable with *T. aestivum* L., enabling the transfer of useful traits into elite cultivars [36]. High yielding synthetic-derived commercial cultivars are being grown extensively in some countries, including, for example, the high yielding Chinese cultivar ‘Chuanmai 42’ [37].

As far as the authors are aware, none of the main genome donors of wheat, or a synthetic wheat line have been tested for ozone tolerance to yield bearing stage. This study aimed to test the following hypotheses: *Ae. tauschii* would be more ozone sensitive than *T. urartu* or *T. dicoccoides*; Synthetic Hexaploid Wheat would have either inherited the ozone sensitivity of the DD genome, or the greater tolerance of the tetraploid AABB genome; and the older wheat cultivar, ‘Maris Dove’, would be less ozone sensitive than the more recent wheat cultivar, ‘Skyfall’. The results suggest that when grown to final yield, *Ae. tauschii* is in fact ozone tolerant, as is primary Synthetic Hexaploid Wheat, with ozone sensitivity found in the AA genome as well as in both the cultivars, though the older cultivar was less sensitive than the more recent one.

## 2. Results

### 2.1. Shoot Biomass

**Figure 2:**
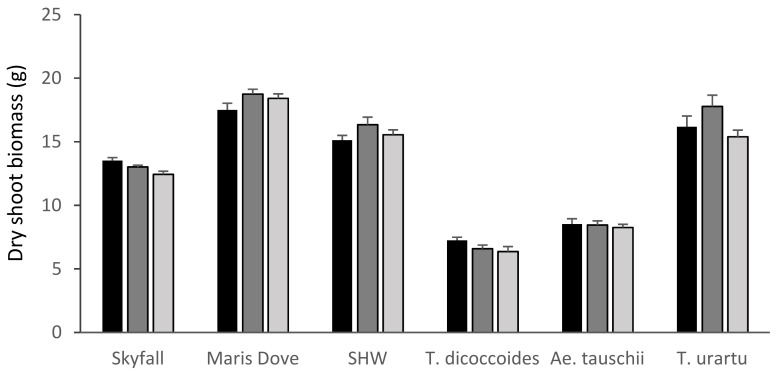
Mean shoot biomass by species and treatment. Skyfall: *T. aestivum* ‘Skyfall’, Maris Dove: *T. aestivum* ‘Maris Dove’, SHW: Synthetic Hexaploid Wheat. Low/30 ppb: black bars; medium/55 ppb: dark grey bars; high/110 ppb: light grey bars. Bars show standard errors (n = 6).

There was no significant overall effect of ozone on the shoot biomass of any species (Figure 2) apart from that of the recently released cultivar ‘Skyfall’ (*p* < 0.001), which showed a significant reduction (*p* < 0.01) in shoot biomass between the low 30 ppb and high 110 ppb ozone levels (−8%) (see Supplementary Materials, Table S1 for all *p* values.). Whilst there was also a reduction in the shoot biomass of *T. dicoccoides* (−12%) and *T. urartu* (−5%), these effects were not statistically significant. The shoot biomass of the line of primary synthetic hexaploid wheat (+3%) and *Ae. tauschii* (−3%) were less affected by ozone than the other varieties tested.

### 2.2. Total Seedhead Biomass

**Figure 3:**
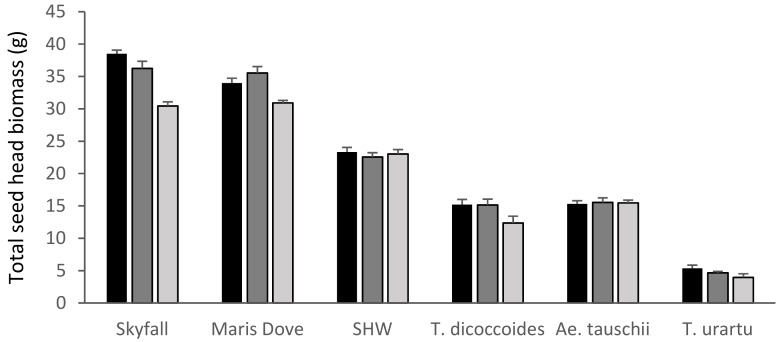
Mean total seed head biomass. Skyfall: *T. aestivum* ‘Skyfall’, Maris Dove: *T. aestivum* ‘Maris Dove’, SHW: Synthetic Hexaploid Wheat. Low/30 ppb: black bars; medium/55 ppb: dark grey bars; high/110 ppb: light grey bars. Bars show standard errors (n = 6).

The cultivars ‘Skyfall (*p* < 0.001) and ‘Maris Dove’ (*p* < 0.01) were the only species where ozone had a significant overall effect on total seed head biomass (Figure 3). The recent cultivar, ‘Skyfall’, showed highly significant reductions in total seed head biomass between the low and high ozone levels (*p* < 0.001, −21%) and also between the medium and high treatments (*p* < 0.001). The older cultivar, ‘Maris Dove’, whilst sensitive to the highest level of ozone, was more tolerant than ‘Skyfall’, with a smaller but still significant reduction in total seed head biomass (*p* < 0.05, −9%) between the low and high treatment levels.

### 2.3. 1000 Grain Weight

**Figure 4:**
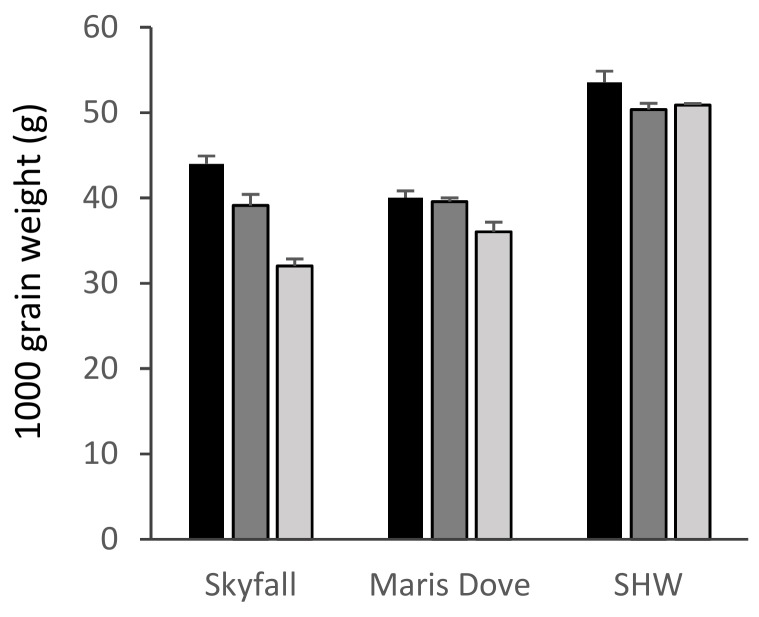
1000 grain weight of cultivar and synthetic wheat. Skyfall: *T. aestivum* ‘Skyfall’, Maris Dove: *T. aestivum* ‘Maris Dove’, SHW: Synthetic Hexaploid Wheat. Low/30 ppb: black bars; medium/55 ppb: dark grey bars; high/110 ppb: light grey bars. Bars show standard errors (n = 6).

**Figure 5:**
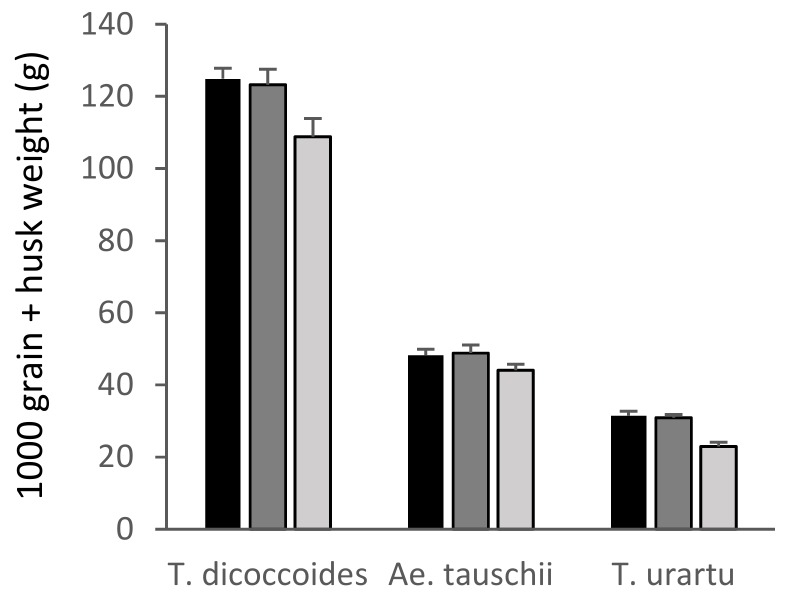
1000 grain + husk weight of the wild relatives of wheat. Low/30 ppb: black bars; medium/55 ppb: dark grey bars; high/110 ppb: light grey bars. Bars show standard errors (n = 6).

There was a significant overall effect of ozone on the 1000 grain/1000 grain + husk weights of all species apart from *Ae. tauschii* (Table S1, Figure 4 and Figure 5). There was a highly significant 27% reduction in the 1000 grain weight of ‘Skyfall’ (Figure 4, *p* < 0.001) between the low and high ozone treatments. The older cultivar ‘Maris Dove’ was less sensitive than ‘Skyfall’, with an 11% reduction between low and high treatments (*p* < 0.05) leading to the 1000 grain weight of ‘Maris Dove’ being greater than that of ‘Skyfall’ under the high ozone treatment. Whilst there was a 5% reduction in the 1000 grain weight of synthetic wheat under both the medium and high levels of ozone compared to the low 30 ppb level, this reduction was only significant between the low and medium levels (*p* < 0.05).

Ozone had a significant negative effect overall on the 1000 grain + husk weight of *T. dicoccoides*, (*p* < 0.05), although the difference between the high and low treatments was not statistically significant (*p* = 0.055) in this parameter. In comparison, *T. urartu* (AA genome) demonstrated ozone sensitivity in its 1000 grain + husk weight, with a highly significant reduction in the high ozone treatments (*p* < 0.001) compared to the low ozone treatment.

These data suggest that *Ae. tauschii* showed ozone tolerance, with ozone having no significant effect on shoot biomass, total seed head biomass or 1000 grain + husk weight (Figure 2, Figure 3 and Figure 5). In contrast, *T. dicoccoides* and *T. urartu* had some degree of sensitivity to ozone, although only *T. urartu* was significantly negatively affected. Across all the parameters measured, the line of primary synthetic wheat appeared more tolerant to ozone than the cultivars, with no significant differences in shoot biomass or total seed head biomass in response to increasing ozone concentration and only a 5% reduction in 1000 grain weight under high ozone (Figure 2, Figure 3 and Figure 4).

## 3. Discussion

### 3.1. Ozone Tolerance in the DD Genome

Based on both shoot biomass and yield data, the accession of *Ae. tauschii* in this study was found to be ozone tolerant. This is contrary to previous assessments of the ozone sensitivity of *Ae. tauschii* using young plants in a short ozone exposure during the vegetative stage [32,33]. The previous studies suggested that *Ae. tauschii* may be the source of wheat’s ozone sensitivity because it was found to have a significantly reduced photosynthetic rate (A_sat_), as well as reduced relative growth rate and shoot and root biomass in response to elevated ozone compared with the other genome donors tested [32]. However, the photosynthetic rate was not determined during the current study.

Wide genetic variation has been found in the wild populations of many *Aegilops* species [30] including *Ae. tauschii* [28]. This variation provides potential for the discovery of novel biotic resistance and abiotic tolerance, but it may also lead to varying responses of different accessions to the same abiotic stress. The discovery that *Ae. tauschii* carries a very high percentage of transposable elements (the DNA sequences that can move within a genome) [38], may explain its ability to adapt to multiple stress factors [28]. The wide geographical range of the *Ae. tauschii* populations means there is potential for multiple novel traits to be accessed, including for a wide range of abiotic stress tolerance [39,40,41].

Prior to the Biswas study, several experiments exposed young wheat plants at the vegetative stage of growth to ozone for 21 days and showed that elevated ozone reduced the mean relative growth rate [21], root:shoot ratios [42] and photosynthetic rates [43]. Based on these studies it was suggested that the effect seen at the vegetative stage may also be reflected in grain yield [22], and this was the rationale behind the Biswas study. However, it is preferable to grow crop plants to final yield to assess ozone sensitivity, partly because yield is the primary indicator of productivity, but also because the effect of ozone has been found to increase as wheat progresses through its growth stages [15] with the greatest degree of ozone damage found to be between anthesis and grain fill [16]. In this study, no data comparative to the Biswas study were collected at the vegetative stage, so no conclusion can be drawn as to whether this accession of *Ae. tauschii* would have also demonstrated ozone tolerance in the early stages of growth.

### 3.2. Ozone Tolerance in Synthetic Wheat

In this study, the line of primary Synthetic Hexaploid Wheat was found to be ozone tolerant. Whilst there was a yield loss of 5% under the high ozone treatment, this was no greater than that under the medium ozone treatment and considerably less than the yield losses of both cultivars. To the authors’ knowledge, there are no studies that have assessed the response to ozone of primary synthetic wheat or cultivars derived from synthetic wheat. However, synthetic wheat has previously been found to provide abiotic stress tolerance [36], with synthetic lines demonstrating tolerance to drought, heat, waterlogging, salinity and frost damage [37,44,45]. It is possible that the abiotic stress tolerance found in synthetic wheat in this trial may relate to beneficial root traits [46], or potentially to higher abscisic acid (ABA) responsiveness [40]. Another causal factor may be that the artificial crossing of the AABB and DD genomes to create synthetic wheat lines has enabled a higher proportion of genetic material from the DD genome of *Ae. tauschii* to be transferred into synthetic wheat compared to hexaploid wheat [28,45]. As *Ae. tauschii* was shown in this trial to be ozone tolerant, this could suggest the DD genome has a role to play in the ozone tolerance of synthetic wheat. A strategic approach to identify the beneficial traits within the *Ae. tauschii* populations has been developed by the National Institute for Agricultural Botany (NIAB) for use in its synthetic wheat breeding programme [47]. Although some studies suggest the derived lines do not necessarily retain the same level of abiotic tolerance as the primary synthetic [36], synthetic lines have been found to have superior abiotic tolerance compared to wheat cultivars, therefore, the screening of lines of synthetic wheat for abiotic tolerance is recommended for future research [35,48]. This would be particularly worthwhile in countries that already grow synthetic-derived cultivars and have high levels of ozone, such as India and China.

### 3.3. Possible Reasons for Ozone Sensitivity in the AA and AABB Genomes

Based on the 1000 grain + husk data, it can be suggested that *T. urartu*, the AA genome, is ozone sensitive and could have contributed to the genetic origin of wheat’s ozone sensitivity. In this study *T. dicoccoides*, the AABB genome was more ozone sensitive than *Ae. tauschii*, but less than *T. urartu*. Higher levels of stomatal conductance can lead to a higher ozone flux and greater sensitivity to ozone if the plant is unable to detoxify [49]. Biswas et al. [32] also found *T. monococcum* (AA genome) to be ozone sensitive, and both *T. monococcum* and *T. urartu* have been found to have high levels of stomatal conductance and photosynthetic rates [32,50,51,52]. Interestingly, in both *T. monococcum* and *T. urartu*, stomatal frequency on the adaxial surface of flag leaves was found to be nearly as high as on the abaxial surfaces [50], which could lead to a greater flux of ozone into the leaf. However, the stomatal conductance of *Ae.tauschii* has also been found to be high [51] and as *Ae. tauschii* was found to be more ozone tolerant in this trial, this is not necessarily the sole factor involved. *T. monococcum* has been found to have a reduced antioxidant capacity, and subsequently, higher levels of reactive oxygen species compared to a wheat cultivar, with cultivated Emmer wheat (*T. dicoccum AABB*) also showing a similar reduced capacity to detoxify [53]. We suspect that both higher stomatal conductance and lower antioxidant levels may be the reasons why both the AA and AABB genomes appeared more ozone sensitive in this study.

### 3.4. Differences in Ozone Sensitivity of the Cultivars

The seed head biomass and 1000 grain weight of the cultivar ‘Maris Dove’ released in 1971, suggested that it was less sensitive to ozone than the more recent cultivar ‘Skyfall’ released in 2014, which had been tested previously and found to be more sensitive than cultivars released in the 1980s and 1990s [54]. These data add to the growing body of evidence that indicates that the more recent cultivars are often more ozone sensitive than older ones [21,22,23]. The exact cause of this variable response between cultivars is still unclear. As higher levels of ozone damage may be correlated with higher levels of ozone flux there is concern that trait selection to increase stomatal conductance in order to maximise photosynthesis [55] may have led to an inadvertent increase in ozone damage [13]. However, a plant’s ability to detoxify through the production of antioxidant enzymes may also play a significant role in ozone tolerance [17], although this factor has not been assessed in relation to the date of release of the cultivar.

The specific genes, or sets of genes, involved in these different ozone tolerance mechanisms are currently unknown but it is worth noting that even though the wheat cultivars and synthetic wheat are both hexaploid wheats, the ozone tolerance trait demonstrated by the line of primary synthetic hexaploid wheat grown in this trial was not present in either of the cultivars. Whilst the AA and BB components of wheat’s hexaploid genome have retained c. 30% of the genetic material from their tetrapoloid wild progenitor *T. dicoccoides*, the DD genome component contains just 10% of the diversity found in *Aegilops tauschii* [56]. It appears that synthetic wheat, with a greater proportion of the DD genome, has more genetic diversity and therefore has, as this trial would suggest, greater potential for abiotic tolerance.

### 3.5. Future Research

Whilst further research is needed to understand the exact causes of wheat’s increasing ozone sensitivity, synthetic wheat and its use in plant breeding may offer an opportunity to develop more ozone tolerant cultivars. Screening for ozone tolerant traits in some wheat cultivars has already taken place [57]. Including synthetic wheat in future screening processes would be a useful next step. The promise that synthetic wheat has shown for ozone tolerance in this trial could be further explored through the assessment of synthetic lines and synthetic derived cultivars, as well as commercial synthetic wheat cultivars already on the market.

## 4. Materials and Methods

### 4.1. Plant Establishment

The experiment was undertaken in 2018 at the Centre for Ecology and Hydrology (CEH) air pollution facility at Abergwyngregyn, North Wales (53.2°N, 4.0°W). The species selected for the trial (Table 1) included a recent high yielding cultivar released in 2014 (*T. aestivum* L., cv. Skyfall), one older cultivar released in 1971 (*T. aestivum* L., cv. Maris Dove), a primary spring Synthetic Hexaploid Wheat line created from a cross between *T. durum* and an accession of *Ae. tauschii*, and three of the main genome donors of bread wheat: *T. dicoccoides*, *T. urartu*, and *Ae. tauschii*. The accession of *Ae. tauschii* was not the same accession used to create the line of synthetic wheat.

De-husked seeds were germinated (11–12 April) in petri dishes in an incubator set at room temperature (21 °C). Seedlings were then sown into modular plug trays containing Levington’s John Innes No. 1 low nutrient seedling compost and grown in a glasshouse without light or temperature control (13–19 April, Bangor, UK). Vernalisation was started 7 days later, for four weeks, (16 h day length; 1.2 klux light intensity; 5 °C) and watered as required. Seedlings were then transplanted (19–21 May) into 6.3 L round plastic pots (25 cm deep, 20 cm diameter at the rim) containing coarsely sieved and well mixed sandy loam soil (originating from Henfaes Research Station, Abergwyngregyn, North Wales), with two seedlings per pot. There were six replicate pots per species and per treatment. Plants were grown in a glasshouse without light or temperature control (22 May–6 June) with pots rotated randomly every 4–5 days. All pots were watered manually, daily or as required, to maintain soil moisture levels throughout the experiment. There was 77 kg ha^−1^ equivalent available nitrogen within the topsoil at the start of the trial. Fertiliser in the form of ammonium nitrate was applied to all species on 21 June after tillering/elongation growth stages with both cultivars and synthetic wheat receiving a rate equivalent to 50 kg ha^−1^, and the wild relative species receiving 25 kg ha^−1^. After anthesis the cultivars received an additional 25 kg ha^−1^ on 26 July, with the only wild relative which was still growing, *T. urartu*, receiving an additional 10 kg ha^−1^ on 1 August.

### 4.2. Ozone Treatment

**Figure 6:**
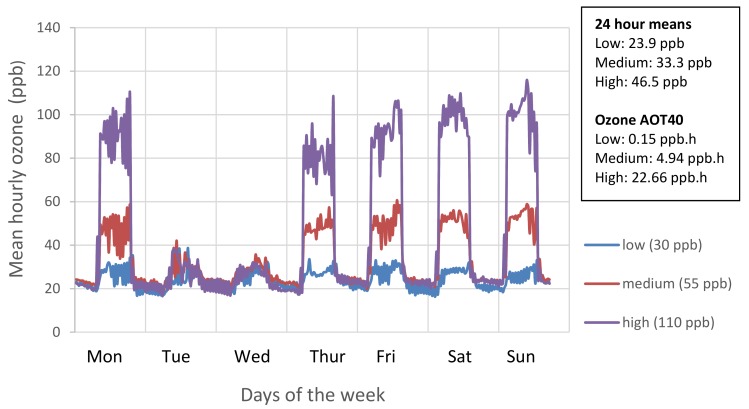
Mean hourly ozone concentrations for the weekly profile achieved over the course of ozone treatment (7 June and 21 August 2018). Seasonal 24-h mean is also shown.

The replicates were distributed randomly within four hemispherical glasshouses (solardomes; 2.1 m high; 3 m diameter). Ozone treatments started on 7th June and continued for 11 weeks until all plants had been harvested (21 August). Using square wave exposures to the maximum levels, ozone concentrations in each solardome provided low (30 ppb), medium (55 ppb), and high (110 ppb) regimes (Figure 6 and Figure S1), with concentrations reduced at night-time and on two days each week, to reflect natural patterns of ozone exposure. Plants were rotated within the solardomes weekly, and once (20 June) between the four solardomes; environmental conditions were found not to vary between the solardomes [58].

Ventilation in the solardomes comprised of approximately two air changes per minute with charcoal-filtered air. Ozone was provided through controlled injection using a G11 ozone generator (Ozone Industries, Andover, UK) and Sequel 10 oxygen concentrator (Pure O2, Urmston, UK), with computer-controlled concentrations (Lab VIEW version 2012, National Instruments, Austin, TX, USA). Ozone was supplied via PTFE tubing, and monitored every 30 min by two calibrated automatic ozone analysers (400a, Enviro Technology Services, Stroud, UK, and Thermo Scientific Model 49i Electron O3 Analyser, Fischer Scientific, Waltham, MA, USA).

### 4.3. Biomass and Yield Measurements

For the cultivars and synthetic wheat, the fully ripened ears were cut from both plants in each pot shortly before harvest. For the wild relatives, seed was either collected by hand prior to shattering (*Ae. tauschii*), or collected through the use of organza bags secured around the seed heads (*T. urartu* and *T. dicoccoides*). As *T. urartu* was still growing at the time of harvest all seed heads were collected, although only ripened seed heads were included in the seed head biomass totals. For all species, both plants from each replicate pot were then harvested by cutting the shoot just above the surface of the substrate. Shoot biomass was obtained by weighing after drying at 65 °C for 14+ days. The ears from each replicate of the cultivars, or the total loose seed collected from each of the wild relative replicates, were weighed to derive the mean total seed head biomass. The ears of the two cultivars and synthetic wheat were threshed using a hand thresher (Minibatt+, Reichhardt Electronic Innovations, Hungen, Germany), and the seeds were weighed. 1000 grain weight was obtained by weighing 100 randomly selected grains from each replicate, then multiplying by ten. For the wild relative species, the seed could not be threshed and 100 grains with husks were selected randomly and weighed, then multiplied by ten to obtain the ‘1000 grain + husk’ weight.

### 4.4. Statistical Methodology

The effect of ozone on the total shoot biomass, total seed head biomass, and 1000-grain weight of each species was assessed using linear models (normal error) and post-hoc Tukey tests in R [59]. Residuals were assessed for normality. All *p* values are listed in the Supplementary Materials (Table S1).

## Figures and Tables

**Table 1:** Wheat and wild relative species used in the trial with details of type and source of seed.

Species/Cultivar	Type	Seed Supplier	Origin/Date
*Triticum aestivum* L., cv. Skyfall	Cultivar	RAGT Semences	Released 2014
	(winter/spring)	France	
*Triticum aestivum* L., cv. Maris Dove	Cultivar (spring)	John Innes Centre Seedstore, Norwich UK	Released 1971
		W0005	
Synthetic Hexaploid Wheat (spring)	100% synthetic wheat	NIAB, Cambridge UKNIAB SHW 084, derived	WISP/Synthetics2011–2017
	(spring)	fromHoh-501(AABB)/Ent-084(DD)	
*Triticum dicoccoides*	Primary wild relative	John Innes Centre Seedstore, Norwich UK	
	AABB genome	T1060020	Israel Collection date not known
*Triticum urartu*	Primary wild relative	John Innes Centre Seedstore, Norwich UK	Turkey Collection date not known
	AA genome	T1010004	
*Aegilops tauschii* (squarrosa)	Secondary wild relative	John Innes Centre Seedstore, Norwich UK	Afghanistan Collection date not known
	DD genome	T2220019	

## Supplementary Materials

The following supplementary materials are available online at https://www.mdpi.com/2223-7747/8/7/195/s1. Table S1: Summary table of *p* values; Figure S1: Weekly ozone concentrations achieved over the course of the plant trial.
