# Supplementary material for: Ozone Tolerance Found in Aegilops tauschii and Primary Synthetic Hexaploid Wheat
Source: Plants (Basel). 2019 Jun 28;8(7):195. doi: 10.3390/plants8070195 (PMC6681361; doi:10.3390/plants8070195)
Supplement: Supplementary file 1 [file plants-08-00195-s001.pdf]

Supplementary Material

Table S1: Table of p values representing levels of statistical significance of the effect of ozone on each species and between the different levels of ozone treatment.

Shoot biomass	Overall effect of ozone by species	LOW – MED	MED - HIGH	LOW - HIGH
cv 'Skyfall'	**0.0084 ** **	0.227	0.165	**0.0062 ** **
cv 'Maris Dove'	0.137	0.131	0.839	0.319
SHW	0.209	0.190	0.476	0.796
T. dicoccoides	0.152	0.326	0.868	0.148
Ae. tauschii	0.843	0.987	0.910	0.839
T. urartu	0.114	0.331	0.103	0.750
Total seed head biomass	Overall effect of ozone	LOW – MED	MED - HIGH	LOW - HIGH
cv 'Skyfall'	**1.017e-05 *** **	0.145	**0.0003 *** **	**< 0.0000 *** **
cv 'Maris Dove'	**0.0018 ** **	0.341	**0.0015 ** **	**0.027 * **
SHW	0.733	0.714	0.884	0.946
T. dicoccoides	0.0699	0.998	0.110	0.101
Ae. tauschii	0.949	0.946	0.992	0.978
T. urartu	0.108	0.446	0.572	0.091
1000 grain weight	Overall effect of ozone	LOW – MED	MED - HIGH	LOW - HIGH
cv 'Skyfall'	**2.574e-6 *** **	**0.011 * **	**0.00057 *** **	**0.0000017 *** **
cv 'Maris Dove'	**0.0074 ** **	0.911	**0.023 * **	**0.0102 * **
SHW	**0.04171 * **	**0.0475 * **	0.901	0.105
T. dicoccoides	**0.03746 * **	0.962	0.073	0.055
Ae. tauschii	0.1828	0.970	0.204	0.291
T. urartu	**0.0001 *** **	0.937	**0.0004 *** **	**0.0002 *** **
**Significance indicated by asterisks where p = < .05 * < .01 ** < .001 *** **				

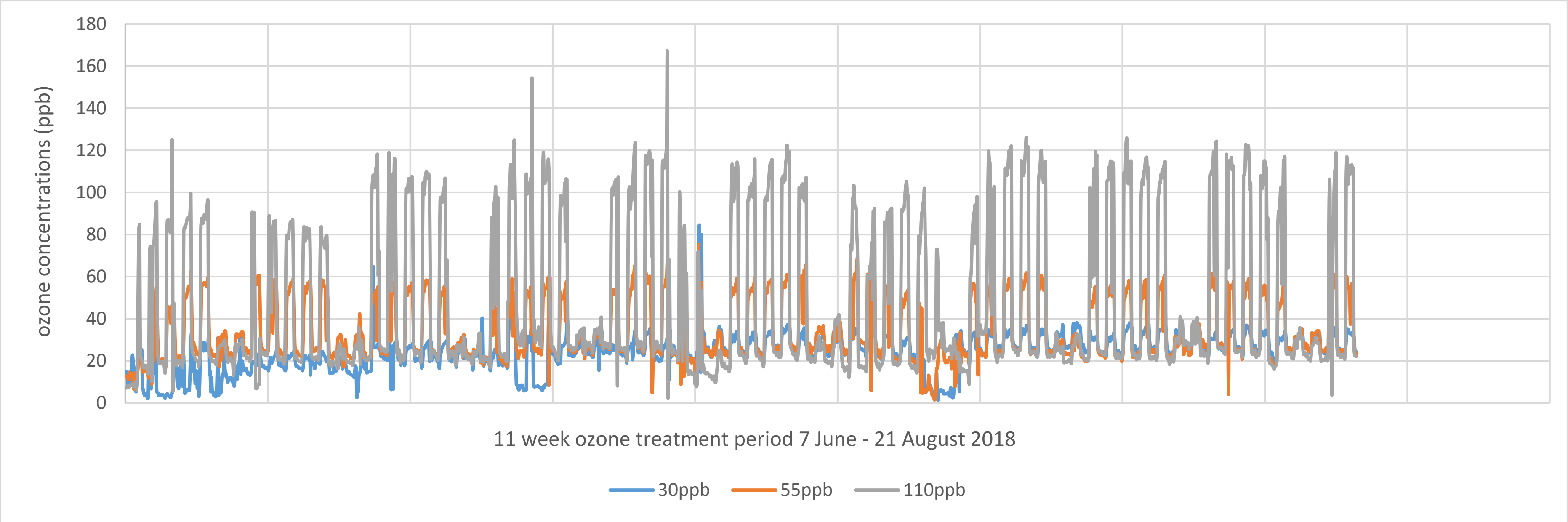

Figure S1 Weekly ozone concentrations achieved over the course of the plant trial.
